# Flood-Exposure Is Associated with Higher Prevalence of Child Undernutrition in Rural Eastern India

**DOI:** 10.3390/ijerph13020210

**Published:** 2016-02-15

**Authors:** Jose Manuel Rodriguez-Llanes, Shishir Ranjan-Dash, Alok Mukhopadhyay, Debarati Guha-Sapir

**Affiliations:** 1 Centre for Research on the Epidemiology of Disasters, Institute of Health and Society, Université catholique de Louvain, Brussels 1200, Belgium; debarati.guha@uclouvain.be; 2 Department of Management, Siksha ‘O’ Anusandhan University, Bhubaneswar 751003, India; mailtosrdash@gmail.com; 3 Tata Trusts, Mumbai 400001, India; 4 Voluntary Health Association of India, New Delhi 110016, India; alok@vhai.org

**Keywords:** flood, disaster, malnutrition, vulnerability, climate change, child, infant, wasting

## Abstract

*Background*: Child undernutrition and flooding are highly prevalent public health issues in Asia, yet epidemiological studies investigating this association are lacking. *Methods*: To investigate to what extent floods exacerbate poor nutritional status in children and identify most vulnerable groups, we conducted a population-based survey of children aged 6–59 months inhabiting flooded and non-flooded communities of the Jagatsinghpur district, Odisha (India), one year after large floods in 2008. Anthropometric measurements on 879 children and child, parental and household level variables were collected through face-to-face interviews in September 2009. The association between flooding and the prevalence of wasting, stunting and underweight was examined using weighted multivariate logistic regression for children inhabiting communities exposed solely to floods in 2008 and those communities repeatedly flooded (2006 and 2008) controlling for parental education and other relevant variables. We examined the influence of age on this association. Propensity score matching was conducted to test the robustness of our findings. *Results*: The prevalence of wasting among children flooded in 2006 and 2008 was 51.6%, 41.4% in those flooded only in 2008, and 21.2% in children inhabiting non-flooded communities. Adjusting by confounders, the increased prevalence relative to non-flooded children in the exposed groups were 2.30 (adjusted prevalence ratio (aPR); 95% CI: 1.86, 2.85) and 1.94 (95% CI: 1.43, 2.63), respectively. Among repeatedly flooded communities, cases of severe wasting in children were 3.37 times more prevalent than for children inhabiting in those non-flooded (95% CI: 2.34, 4.86) and nearly twice more prevalent relative to those flooded only once. Those children younger than one year during previous floods in 2006 showed the largest difference in prevalence of wasting compared to their non-flooded counterparts (aPR: 4.01; 95% CI: 1.51, 10.63). Results were robust to alternative adjusted models and in propensity score matching analyses. For similar analyses, no significant associations were found for child stunting, and more moderate effects were observed in the case of child underweight. *Conclusions*: Particularly in low-resource or subsistence-farming rural settings, long-lasting nutritional response in the aftermath of floods should be seriously considered to counteract the long-term nutritional effects on children, particularly infants, and include their mothers on whom they are dependent. The systematic monitoring of nutritional status in these groups might help to tailor efficient responses in each particular context.

## 1. Introduction

In the last decade alone, floods have affected nearly one billion people worldwide [1]. Despite their potential burden to society, the consequences of floods on human health remain rarely investigated, and the few studies that do suggest increased short-term risks of mortality, injury, certain communicable diseases and psychosocial trauma [2,3,4]. Our understanding of the long-term health consequences of flooding and recurrent floods is even more limited [2,3,5]. Moreover, the impacts of flooding on nutritional health are likely to be quite severe among young children in developing countries [6,7,8]. Multiple mechanisms explaining how floods may lead to poor nutrition in these settings have been proposed [9,10].

Floods can severely disrupt livelihoods, especially in low-resource settings [7,8]. Flooded households are affected by a plethora of adverse conditions including food insecurity due to crop failure or food affordability due to sudden price changes [7,9]. Daily care of children and breastfeeding practices is importantly challenged during floods as in worst scenarios all basic services become disrupted, including water and sanitation conditions, or the provision of community basic health and social services [7,8,9,10,11]. In addition, diarrhea or respiratory diseases, which occur at increased rates during or after flooding [2,3] have the potential to worsen child nutritional status [11]. 

Notably, most of the global burden of child undernutrition is clustered in south Asia and sub-Saharan Africa [11]. In Asia alone, Black *et al*., estimated 96 million cases of stunted children (those presenting growth failure) and additional 36 million of wasting cases (abnormal thinness indicating a process of weight loss) in children younger than five in 2011 [11]. Moreover, Asia is particularly prone to flood hazards [12]. In 2011 alone, nearly 130 million people were affected by flooding [1], of which at least 11 million might be estimated children under five [13]. 

These nutritional problems in children might be seriously aggravated by the augmented likelihood of extreme precipitation events and flooding due to climate change [14] but also by increasing social and physical vulnerability to flood hazards [15]. In particular, children’s higher susceptibility to climate-related hazards [16] coupled with climate-related food insecurity in many developing economies [17] might increase the burden of disease due to flooding, jeopardizing human development of future generations [11].

Sound evidence on the association of flooding and child nutrition is still rare and population-based studies are lacking [2,3]. Despite national and international NGOs, and international health organizations have claimed for years the consequences of floods on nutritional health of the most vulnerable, far-reaching change on both tailored prevention and response policies will require sound scientific evidence. 

The state of Odisha, surrounding the Bay of Bengal, is prone to numerous hazards, such as floods, droughts or cyclones, but the floods that occurred in September 2008 were unprecedented in their impact [18]. Overall, 4.5 million people were affected by flooding, with severe losses and disruption of fishing activities, livestock, silk and crop farming. Drinking water supplies were importantly affected by the flood. Schools, front-line care and social services were challenged to operate normally [19]. Anganwadi centres were also damaged. These are important community resources, as they are used as shelter in case of disaster and provide service to 0–6 year old children and pregnant and lactating mothers under the Integrated Child Development Scheme (ICDS). The government provided emergency relief packages among the affected populations, including food and basic utilities to children and adults, covering their basic needs for the first 15 days following the floods [18].

Here we sought to examine the association between flooding and child undernutrition in an epidemiological, controlled rural population-based study representative of 17,876 children aged 6 to 59 months conducted in September 2009. We assessed children affected solely by the 2008 flood and by repeated flooding in 2006 and 2008 and investigated the most vulnerable child cohorts through the interaction of flood exposure with child’s age.

## 2. Materials and Methods

### 2.1. Analysis Plan

Our study focused on the differences in relevant anthropometric indicators (*i.e.*, stunting and wasting) between flooded and non-flooded populations. Our second research objective reviewed these differences for the prevalence of moderate and severe undernutrition indicators. We additionally provided population-weighted estimates of the prevalence of stunting, wasting and underweight in the overall, flooded and non-flooded surveyed populations. We investigated the nutrition of child cohorts affected by floods in 2006 and 2008, and those children affected only by floods in 2008. An age interaction was examined to investigate the most vulnerable age groups to the effects of floods, as this proved to be relevant on a previous study [7].

As our primary aim was to estimate the effect of flooding on child undernutrition, we excluded variables (e.g., caregiving practices, food security, and access to health care, water and sanitation, diarrheal morbidities) which may have mediated this effect [20]. We supported this selection by examining the data collected on these variables framed within the UNICEF conceptual malnutrition framework [21]. Overall, 51 variables were assessed. Out of these, 18 plausible mediators were excluded. The remaining 33 variables were assessed as possible confounders [22]. We also tested if the frequencies of the 33 variables varied across flooded and non-flooded communities [23]. Two adjusted models were proposed, one controlling for confounders, another adjusting for confounders in addition to other variables which differed across exposure. 

### 2.2. Exposure Data

The list of flooded villages during the 2006 and 2008 events in the four selected blocks was obtained from the Odisha State Disaster Mitigation Authority, OSDMA [24]. The estimated percentage of affected population within each of the 122 villages was also provided. Exposure to flooding was defined as living in a village flooded in 2008. Overall, nearly 90% of the population was estimated as directly affected by the floods in these villages (mean = 92.5%; SEM = 1.5). An additional 143 villages were unaffected by this flood and located in the same blocks, and were used as the comparison group. For the 2006 flood event, OSDMA reported 110 villages as flooded in the same four blocks, with a majority of these (*n* = 101, 92%) also affected in 2008. The data was provided as the estimated percentage of the population affected by the floods within each village. This was used in controlled analyses but did not affect the study design.

### 2.3. Study Design and Participants

Jagatsinghpur is a coastal district located in the state of Odisha, eastern India. It has a population of one million with around 90% residing in rural areas [25]. The region is located in a large flood plain which is part of the Himalayan system and is subject to heavy monsoons and recurrent flooding. The district has been severely hit by five major floods in the last decade, the one following the cyclone Paradip (05B) in 1999, followed by heavy floods in 2001, 2003 and 2006. In our study area, villages have been only affected by 1999 cyclone Paradip before the 2006 floods. The latest floods, starting in mid-September 2008, produced great devastation [1,18]. 

We used a population-based cross-sectional design to estimate the association of exposure to flooding in 2008 with the prevalence of child undernutrition one year later in rural Jagatsinghpur district, Odisha, India. A two-stage cluster survey was required to obtain a probability sample of 900 children aged 6 to 59 months representing children of this age in 265 villages located in four severely flood-affected blocks of the district (Kujang, Biridi, Balikuda, Tirtol). Of these, 122 have been flooded and 143 non-flooded in September 2008. The percentage of households flooded in each village was obtained for 2006 and 2008 events from OSDMA. 

**Figure A1:**
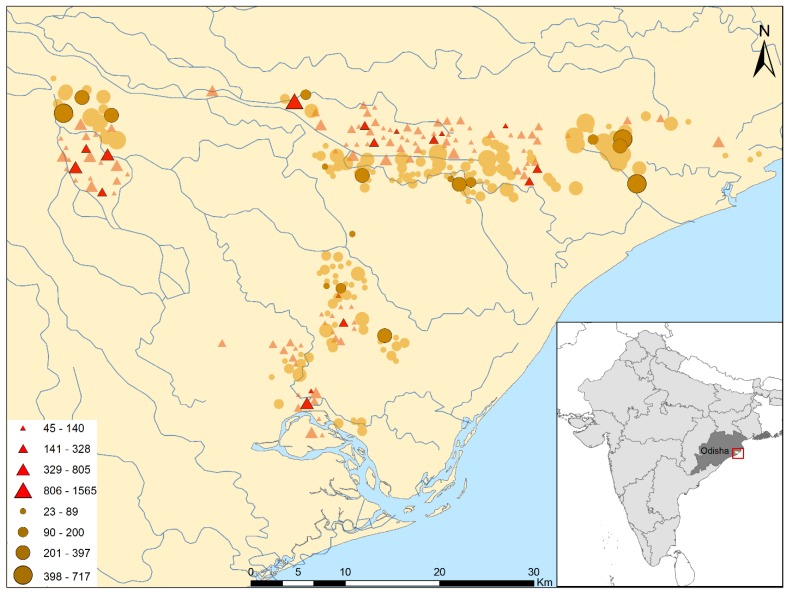
Study site, eligible villages and sample of flooded and non-flooded villages in Jagatsinghpur district, Odisha, India. Triangles represent flooded villages; circles those non-flooded. The size of triangles and circles is approximately proportionate to village size measured by number of households (see map legend). Polygons overimpressed identified villages selected by PPS.

At the first sampling stage, 30 clusters (29 villages) were selected with probability proportional to their size. At the second sample stage we did a census on children living in each selected village which allowed us to randomly select children. This design is useful if the population of interest is clustered in villages and the information on the population itself is limited [26]. The Primary Sampling Units (PSUs) were the villages and the Secondary Sampling Unit (SSU) were the children. We considered a 30 (PSU) by 30 (SSU) design, which should provide a probability sample of 900 children. We firstly listed all 265 villages with their population size projected for 2009 from the available census [24]. Subsequently we selected 30 clusters (29 villages) out of the 265 listed with probability proportional to its population. Village population size ranged between 7 and 9430 inhabitants. Probability Proportionate to Size (PPS) sampling with replacement (e.g., one large village was selected twice) was used to select villages with unequal size and give an equal probability to each child in the population of being selected [26]. Second, within each selected village an updated list of eligible children (*i.e*., a 6 to 59 month old child living in the surveyed village at time of interview) was obtained from the ICDS centers (1 ICDS center for every 1000 inhabitants) and validated with the ward members of each village. In all 3,671 eligible children were listed from 29 villages during the month preceding the training and piloting of the questionnaire. Once the lists were compiled, it was detected that three flooded villages (Korana, Jamphar and Raghunathpur) and two non-flooded (Muthapada, Sureilo) did not have enough eligible children (less than 30). We therefore created five new clusters by merging the list of children in each of these five villages (*n* = 280) with those of the closest non-selected flooded or non-flooded village, respectively, of our list (Figure A1). At the second stage of the sampling, thirty children per cluster were randomly selected.

At the time of planning this research in April–June 2009, the only available information on the children population was that of the Indian census of 2001 [27]. We estimated the total population and number of children living in these 265 villages using state-level population projections for 2009 available at the census [27]. The total population living in these villages was estimated at 228,550. Using this method, we initially calculated that the survey would represent a population of roughly 17,876 children aged 6 to 59 months living in flood-prone areas of Odisha. 

The sample size required to answer our research question was calculated *a priori* using OpenEpi [28]. Based on results from a previous study [7] we set a 15% difference in prevalence of undernutrition, considering the prevalence in flooded communities would be 50% (35% in non-flooded). Setting a prevalence to 50% in the power calculation provides the largest sample size possible for any combinations given the prevalence difference is constant, and thus provides conservative samples. For a power of 80% with α (error) = 0.05 and approximately equal samples of the compared groups, a sample of 340 children (170 in each group) was required. We used a conservative design effect of 2 to adjust for a complex survey design. Overall, 680 children (340 in each group) were required to safely answer our research question. Accounting for maximum attrition in our sample of 25%, we planned to sample 900 children.

### 2.4. Ethical Considerations

Ethical approval was provided by the Community Health Ethics Committee, Voluntary Health Association of India, New Delhi. All the participants involved in the study were informed about the nature of the study, research objectives and about confidentiality of the data, with the assurance that non-participation would not lead to negative consequences. Persons eligible to participate in the study were not offered a monetary incentive for participation. Written informed consent was obtained for every head of household visited. In case the respondent was illiterate, we asked a literate person from the community to read out the consent form and explain it to the head of the family. Then we obtained the thumb impression of the respondent. In those cases, the person who read the consent form also signed as a witness. Research procedures were consistent with the Declaration of Helsinki [29]. Interviews were administered after obtaining informed consent. The protocol was reviewed by a small group of scientists who had experience working with survivors of natural disasters and amended based on their recommendations.

### 2.5. Procedures

We used three anthropometric indicators to assess malnutrition: stunting or chronic malnutrition expressed as height-for-age, underweight as weight-for-age and wasting or acute malnutrition as weight-for-height. Weight measurements were undertaken to the nearest 100 g by trained research assistants using a beam balance (<10 kg; Raman Surgical Co., Delhi, India) and an electronic balance for those children heavier than 10 kg. For children younger than 2 years of age, length was measured to the nearest millimeter in the recumbent position using an infantometer (Narang Medical, Delhi, India). Children older than 2 years were measured in standing position using an adjustable board calibrated in millimeters. We measured and weighed each child twice to minimize measurement errors and use the average value of both measurements to gain precision. All instruments were calibrated daily.

The survey questionnaire used in this study was adapted from the core one developed and approved by a multidisciplinary consortium of researchers (*i.e.*, sociologists, psychologists, epidemiologists, economists and public health scientists) working as part of the MICRODIS project. The instrument development was based on interim literature reviews, and closely followed the UNICEF conceptual framework on child malnutrition [21]. A survey questionnaire equivalent to ours was successfully used in another site in India. The questionnaire was used to collect background information at the household level, and more specifically from mothers and fathers, covering basic socio-demographic characteristics, wealth, child caring practices, healthcare access, maternal and paternal education, income and credit practices, water and sanitation, food consumption patterns; demographics, nutrition and health status data at the child level.

The questionnaires were administered by the Voluntary Health Association of India (VHAI), a non-profit organization which supports social and health-related country-wide initiatives. Twelve experienced research assistants [7] received a three-day training on anthropometry and interview procedures in late August 2009. The questionnaire was piloted in 12 households (six in flooded villages and six in non-flooded) and revised based on the pilot exercise. The final questionnaire was translated to the local language in Odisha (Oriya) and then back translated into English by different professional translators and a researcher checked agreement between both versions. All interviews lasted 45–60 min. Field work was carried out between the 6th and 24th of September 2009. 

### 2.6. Statistical Methods

ENA for SMART software (version November 2008) was used to calculate nutritional indicators with the 2006 World Health Organization Standard [30]. Missing data was rare: ten children with ages out of range were excluded as well as two children with missing values for weight-for-height. Children from five additional villages not in the original PPS selection were not included in the final analysis as our study sample provided enough power to answer our main research question.

We examined the relationship between flood exposure and undernutrition in unadjusted and multiple adjusted logistic regression models with a quasi-binomial distribution to control for overdispersion [23]. Confounders were variables associated with child nutrition statistically, having a *p*-value equal or lower than 0.2 starting with a full model using 33 relevant variables [22]. Backward selection was used to get the final list of confounders for each nutritional indicator. At each step the non-significant variable with the largest *p* value was excluded to obtain a final model including significant variables (*i.e.*, *p* ≤ 0.2). Weighted Wald tests and *t*-tests (in the particular case of testing mean differences) were used to compare each variable distribution across flooded and non-flooded populations [23], with a significance level alpha of 0.2.

Results were provided as prevalence ratios, crude (PR) or adjusted (aPR) with 95% confidence intervals. The two-way interaction of flood-exposure with the variable child age was examined. All tests were two-tailed with α = 0.05. All analyses were weighted. Weights were calculated as the inverse of the selection probabilities. Statistical analyses were conducted in R (Version 3.0.2) [31] with the survey package [23]. To check for population differences across exposure or potential selection bias, propensity score analysis was carried out with the package MatchIt [32]. The nearest neighbor method was performed to match samples in flooded and non-flooded cohorts by similar covariates distributions as indicated by calculated propensity scores [32]. Reporting of the study followed the STROBE guidelines (Table B1).

### 2.7. Estimates of Population Size

Through our survey data, we assessed the total children population aged 6 to 59 months living in these 265 villages of Odisha. A total 16,791 children (95% CI: 15,979, 17,602) were estimated, with 6810 children in the 122 flooded villages (95% CI: 6128, 7493) and 9,980 inhabiting the 143 non-flooded villages (95% CI: 9043, 10,917). These estimates provide an idea of the population size to which the subsequently presented findings apply.

## 3. Results

### 3.1. Sample and Determinants of Child Undernutrition in Flooded and Non-Flooded Communities

**Figure 1:**
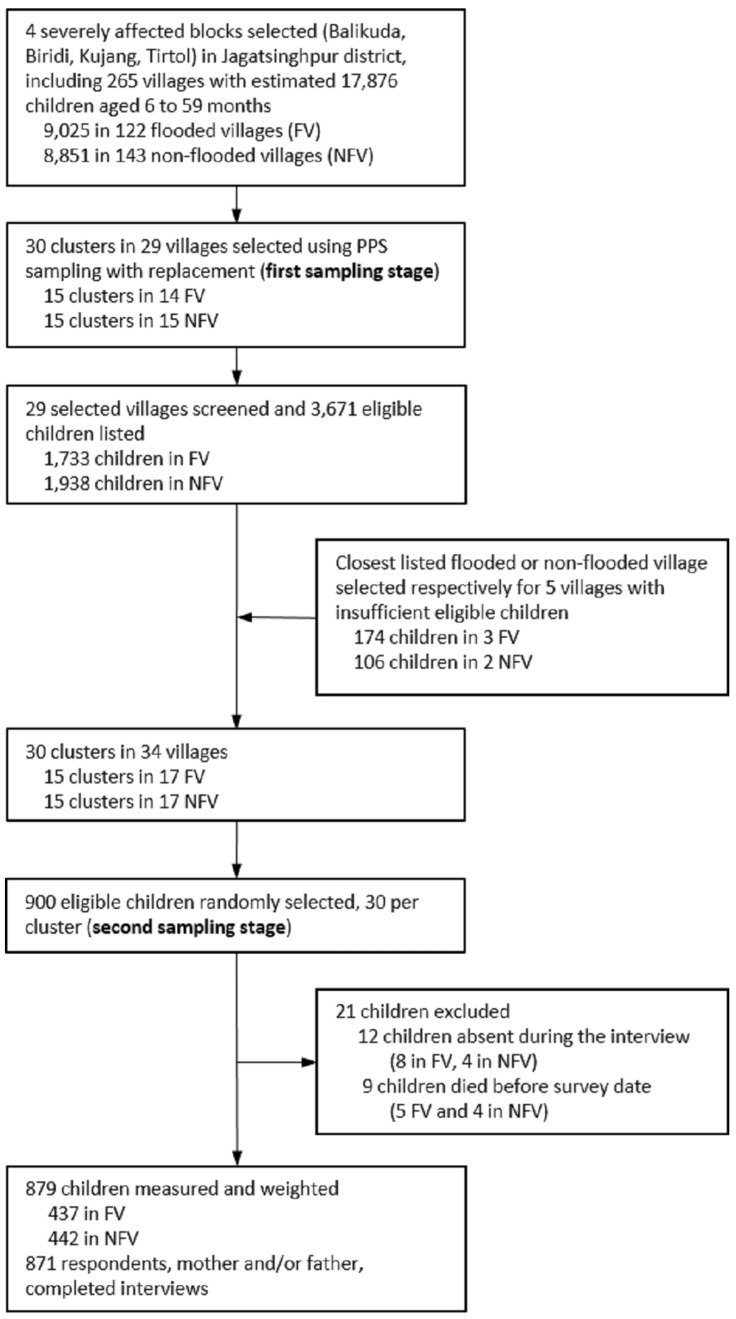
Flow diagram of sample selection.

Out of 900 selected children, 21 could not be measured and were not replaced. Out of these, 12 were not present at the time of the interview and were not found in the two additional visits to their households. Of these cases, eight occurred in the flooded villages and the other four in the non-flooded. In addition, nine children died within the period between the collection of the lists and the day they were surveyed. Out of these children, five lived in flooded villages and the remaining four in non-flooded (Figure 1). The overall response rate was 97.7% and consistent across flooded (97.1%) and non-flooded (98.2%) children’s parents.

Baseline characteristics of mothers, fathers, children and households are presented in Table 1 and are classified according to broad categories in the UNICEF conceptual malnutrition framework [21]. Overall, the distributions of a child’s age, the mother’s age at marriage and at first delivery, breastfeeding and complementary food practices, mother hand washing practices (*i.e.*, before feeding the child and before preparing food for the family), improved latrine, improved toilet, maternal education, paternal education, caste and owned land, all differed across flooded and non-flooded children populations.

### 3.2. Effect of Floods on Child Undernutrition

Overall, the prevalence of wasting was slightly higher in children of flooded communities in 2006 and 2008 (Prev: 51.6; 95% CI: 45.0, 58.2) compared to those living in villages flooded in 2008 (Prev: 41.4; 95% CI: 26.4, 56.4) but especially relative to those non-flooded (Prev: 21.2; 95% CI 15.8, 26.6). Crude and adjusted models showed consistent effect sizes for the associations (Table 2). Flooding was most significantly associated with wasting indicators, not with stunting and smaller effects, although statistically significant, were observed for its association with underweight.

In models adjusted by confounders (Table A1), children repeatedly exposed to flooding in 2006 and 2008 showed slightly larger aPRs for total wasting (2.30; 95% CI: 1.86, 2.85) but comparable to 1.94 (95% CI: 1.43, 2.63) observed in those flooded exclusively in 2008. However, children additionally exposed to floods in 2006 had more than a 3-times higher prevalence of severe wasting relative to those non-flooded (aPR: 3.37; 95% CI: 2.34, 4.86) and nearly doubled that of those only exposed to 2008 floods. The prevalence of moderate wasting was similar across the two exposed groups (Table 2).

### 3.3. Effect of Age on Undernutrition

Table 3 shows that age is an important covariate to consider in the final model, but only to model the children in communities repeatedly flooded. For those children, our analyses revealed specific age vulnerabilities related to previous flood experience. In particular, the cohort of children younger than one year at times of previous floods in 2006 (in our study aged 36–47 months) were those presenting the largest difference in prevalence of wasting compared to their non-flooded counterparts (aPR: 4.01; 95% CI: 1.51, 10.63; *p* = 0.005).

Propensity score analysis was conducted on two large sets of covariates to corroborate these findings. The two additional analyses were consistent with our results (Table A2).

**Table 1:** Determinants of child undernutrition in flooded (in 2006 and 2008, or 2008) and non-flooded communities of rural Jagatsinghpur district, Odisha, India in September 2009.

Variables	Flooded (*n* = 370)		Non-Flooded (*n* = 394)		
	***n***	**% (95% CI)**	***n***	**% (95% CI)**	***p***
**Child characteristics**					
Child female	171	44.8 (38.7, 50.8)	179	44.9 (39.1, 50.1)	0.97
Mean birthweight, g ^1^	370	2783.6 (2734.3, 2832.9)	394	2748 (2707.2, 2788.7)	0.28
*Child age, months*					0.16
6–11	38	8.5 (5.8, 11.2)	47	12.0 (8.4, 15.5)	
12–23	85	25.1 (19.5, 30.7)	100	24.3 (19.4, 29.3)	
24–35	74	19.7 (15.0, 24.5)	81	21.4 (16.3, 26.5)	
36–47	89	24.6 (19.2, 30.0)	69	17.1 (13.0, 21.2)	
48–59	84	22.0 (17.0, 27.1)	97	25.2 (20.0, 30.5)	
**Immediate Causes**					
*Dietary intake*					
Household did experience food shortage due to 2008 flood	314	86.4 (82.4, 90.3)	56	5.6 (3.3, 7.9)	<0.0001
Mean number of daily meals (last week) ^2^	370	2.07 (2.04, 2.09)	394	2.05 (2.02, 2.08)	0.37
Mean weekly vegetable consumption (last week) ^3^	370	7.7 (7.3, 8.0)	394	7.5 (7.1, 7.8)	0.40
*Child’s disease*					
Diarrhea (last two weeks)	127	33.5 (27.9, 39.2)	113	28.2 (22.8, 33.5)	0.18
Respiratory symptoms (last two weeks)	207	58.6 (52.7, 64.6)	199	47.3 (41.4, 53.1)	0.007
**Underlying Causes**					
*Food insecurity (related to flood in 2008)*					
Crop washed away	157	48.9 (42.8, 55.1)	0	0.0 (0.0, 0.0)	<0.0001
Lack of earning opportunities	50	13.5 (9.7, 17.3)	0	0.0 (0.0, 0.0)	<0.0001
Increase in food price	37	7.9 (4.9, 10.9)	9	2.0 (0.7, 3.4)	<0.001
Grain stock washed away	26	5.9 (3.5, 8.3)	0	0.0 (0.0, 0.0)	<0.0001
Food unavailable in market	26	5.6 (3.4, 7.8)	15	3.6 (1.7, 5.4)	0.17
Household members migrated	16	4.1 (2.1, 6.1)	0	0.0 (0.0, 0.0)	<0.0001
*Care of mother and children*					
Mean age at marriage of the mother, y ^4^	370	21.9 (21.6, 22.3)	394	21.5 (21.2, 21.9)	0.10
Mean age of the mother at first delivery, y ^5^	370	23.7 (23.4, 24.1)	394	23.4 (23.1, 23.8)	0.19
Mean age of the mother at birth of child, y ^6^	370	26.1 (25.7, 26.5)	394	25.9 (25.5, 26.3)	0.55
Mean age of the father at birth of child, y ^7^	370	31.3 (30.8, 31.8)	394	31.1 (30.6, 31.7)	0.61
Two or more children underfive eating from same kitchen	142	38.0 (32.1, 43.9)	168	39.8 (34.2, 45.4)	0.66
Mean number of child visits to ICDS (last month) ^8^	370	1.2 (0.9, 1.5)	394	1.2 (0.9, 1.5)	0.96
Mother was a member of self-help group/Mahila Mandal	68	17.8 (13.6, 22.0)	70	17.1 (13.0, 21.2)	0.82
Mean number of mother’s conceptions ^9^	370	1.9 (1.8, 2.1)	394	2.0 (1.9, 2.1)	0.51
Mother had one or more miscarriages, stillbirths	50	12.8 (9.0, 16.7)	61	15.5 (11.1, 19.9)	0.37
Child received deworming treatment in past 6 months	132	35.8 (29.9, 41.6)	145	37.3 (31.5, 43.0)	0.72
Child breastfed at least 2 years	123	31.9 (26.3, 37.5)	131	35.0 (29.3, 40.7)	0.65
Supplementary feeding started after 6 months of child’s age	251	66.8 (60.9, 72.8)	265	68.7 (63.3, 74.0)	0.65
Child breastfed at least 2 years and supplemented after 6 months of age	81	20.7 (15.9, 25.4)	98	26.7 (21.3, 32.1)	0.10
Mother washes hands with soap before feeding child	72	17.5 (13.1, 21.9)	82	22.7 (17.3, 28.0)	0.14
Mother washes hands with soap before preparing food for the family	51	12.4 (8.5, 16.3)	68	19.5 (14.2, 24.7)	0.035
Mother washes hands with soap before lunch	71	18.5 (13.9, 23.2)	80	21.9 (16.5, 27.2)	0.36
Mother washes hands with soap after attending the child who defecated	121	29.2 (23.9, 34.5)	121	31.9 (26.1, 37.6)	0.51
Child received BCG vaccine	254	66.4 (60.4, 72.3)	274	68.4 (62.8, 74.1)	0.62
Child received measles vaccine	224	58.8 (52.7, 64.9)	232	57.9 (52.1, 63.8)	0.84
Child received all three doses of polio vaccine	245	64.4 (58.4, 70.4)	260	65.9 (60.2, 71.6)	0.73
Child received all three doses of B-hepatitis vaccine	41	10.6 (6.9, 14.4)	35	8.9 (5.8, 12.1)	0.50
Child received all three doses of DPT vaccine	247	64.8 (58.8, 70.8)	261	66.1 (60.4, 71.8)	0.76
*Public health*					
Improved latrine (before floods)	126	32.6 (26.7, 38.4)	341	86.4 (81.9, 90.9)	<0.0001
Improved latrine (after floods)	58	13.3 (9.4, 17.2)	341	86.4 (81.9, 90.9)	<0.0001
Improved drinking water (before floods)	361	98.2 (97.0, 99.4)	341	86.7 (83.0, 90.3)	<0.0001
Improved drinking water (after floods)	232	60.6 (54.5, 66.8)	NA	NA	NA
Mother gave birth at hospital	306	84.2 (80.1, 88.4)	324	82.4 (78.2, 86.6)	0.54
Child received treatment at private medical consultation, community healthcare or hospital of diarrhea or respiratory symptoms	160	44.8 (38.6, 50.9)	178	43.1 (37.4, 48.9)	0.70
*Number of individuals eating from same kitchen*					0.15
<5	69	19.7 (14.6, 24.7)	103	25.2 (20.2, 30.2)	
≥5 and <6	55	14.4 (10.0, 18.8)	75	18.3 (14.1, 22.4)	
≥6 and <8	121	32.2 (26.7, 37.8)	99	29.2 (23.4, 35.1)	
≥8	125	33.6 (27.8, 39.5)	117	27.3 (22.4, 32.3)	
*Capital*					
Mean annual income *per* household Capita ^10^	370	7642.6 (6582.3, 8702.9)	394	7554.4 (6316.1, 8792.6)	0.40
Household did borrow money (loan, credit, micro-credit)	31	8.1 (4.9, 11.2)	22	5.2 (3.0, 7.4)	0.70
*Maternal education*					0.011
None	20	5.0 (2.8, 7.2)	37	9.2 (5.8, 12.7)	
Primary school	64	16.7 (12.2, 21.2)	96	22.7 (18.1, 27.4)	
Middle school	126	35.3 (29.3, 41.2)	105	25.2 (20.5, 29.9)	
High school	116	32.1 (26.2, 38.0)	110	28.4 (23.1, 33.7)	
College or more	44	10.9 (7.4, 14.5)	46	14.4 (9.4, 19.5)	
*Paternal education*					0.014
None	7	2.2 (0.5, 4.0)	15	4.0 (1.3, 6.6)	
Primary school	32	7.3 (4.6, 10.0)	55	13.8 (9.8, 17.7)	
Middle school	103	24.9 (20.0, 29.8)	116	28.6 (23.5, 30.7)	
High school	143	40.7 (34.6, 46.8)	123	29.9 (24.7, 35.0)	
College or more	85	24.8 (19.3, 30.4)	85	23.8 (18.3, 29.3)	
*Land ownership, hectare*					0.003
<0.04	74	17.1 (13.0, 21.2)	115	29.7 (24.1, 35.3)	
≥0.04 and <0.2	84	21.1 (16.4, 25.9)	89	21.9 (17.2, 26.5)	
≥0.2 and <0.8	92	26.6 (21.0, 32.3)	98	23.2 (18.7, 27.8)	
≥0.8	120	35.2 (29.2, 41.2)	92	25.2 (19.9, 30.6)	
Household did own any livestock or poultry	236	64.7 (58.9, 70.6)	213	55.2 (49.3, 61.0)	0.024
**Basic causes**					
*Religion*					0.55
Hindu	318	91.9 (89.6, 94.1)	366	93.0 (90.1, 95.8)	
Muslim	52	8.1 (5.9, 10.4)	28	7.0 (4.2, 9.9)	
*Caste*					0.001
General	116	38.4 (32.1, 44.7)	73	22.2 (16.8, 27.5)	
Other backward	134	33.7 (28.2, 39.2)	186	41.3 (35.7, 47.0)	
Scheduled caste	69	20.1 (15.2, 24.9)	107	29.5 (24.0, 35.0)	
No caste	51	7.9 (5.7, 10.1)	28	7.0 (4.2, 9.9)	

^1–10^ Mean difference between flooded and non-flooded subpopulations using weighted *t*-test. Wald tests were used in all other comparisons.

## 4. Discussion

Approximately one year after the 2008 flood event, the prevalence of wasting in both flooded and non-flooded populations were well above the 15% prevalence consider as critical threshold by WHO [33]. Our final analyses showed that children living in villages repeatedly flooded presented over 2-fold increased prevalence of wasting compared to those never flooded. Overall, children flooded twice had a prevalence of severe wasting more than three times higher compared to those non-flooded and almost doubled that of those flooded only once. Finally, those children younger than one year when previous floods struck Odisha in August 2006, showed on average a prevalence of total wasting four times higher than those children inhabiting intact villages.

To our knowledge, this is one of the first epidemiological study to provide population-based estimates on the association of flooding and child undernutrition. Controlling for exposure to previous disasters (*i.e*., in this case the 2006 flood) is a rare feature in related literature but essential to ascertain the effects of flooding on child nutritional outcomes. 

**Table 2:** Adjusted and crude associations of repeated exposure to flooding (2006 and 2008) and single exposure in September 2008 with the prevalence of wasting, stunting and underweight as at September 2009 amongst 6 to 59 months children in rural Jagatsinghpur district, Odisha, India.

		Flooded Twice (*n* = 303)		Flooded Once (*n* = 67)		Non-Flooded (*n* = 394)		Adjusted Model 1		Adjusted Model 2		Unadjusted model	
**Indicator**	**Variable**	***n***	**% (95% CI)**	***n***	**% (95% CI)**	***n***	**% (95% CI)**	**Twice *vs.* None aPR (95% CI)**	**Once *vs.* None aPR (95% CI)**	**Twice *vs.* None aPR (95% CI)**	**Once *vs.* None aPR (95% CI)**	**Twice *vs.* None PR (95% CI)**	**Once *vs*. None PR (95% CI)**
Wasting (weight-for-height, WHZ)													
	Moderate	93	30.5 (24.5, 36.6)	22	31.3 (17.0, 45.6)	42	14.3 (9.2, 19.4)	2.06 (1.43, 2.97)	2.22 (1.42, 3.48)	2.04 (1.29, 3.24)	2.23 (1.39, 3.57)	2.14 (1.42, 3.21)	2.19 (1.23, 3.91)
	Severe	63	21.1 (15.9, 26.3)	9	10.1 (3.3, 16.8)	28	6.9 (4.2, 9.6)	3.37 (2.34, 4.86)	1.76 (0.83, 3.72)	3.06 (1.79, 5.21)	1.46 (0.64, 3.33)	3.04 (1.92, 4.81)	1.45 (0.67, 3.13)
	Total	156	51.6 (45.0, 58.2)	31	41.4 (26.4, 56.4)	70	21.2 (15.8, 26.6)	2.30 (1.86, 2.85)	1.94 (1.43, 2.63)	2.36 (1.73, 3.20)	1.93 (1.32, 2.82)	2.43 (1.83, 3.23)	1.95 (1.25, 3.04)
Stunting (height-for-age, HAZ)													
	Moderate	58	20.1 (14.6, 25.6)	9	9.4 (3.1, 15.8)	75	17.2 (13.0, 21.4)	1.03 (0.73, 1.47)	0.47 (0.29, 1.09)	0.92 (0.57, 1.47)	0.45 (0.20, 1.04)	1.17 (0.81, 1.69)	0.55 (0.27, 1.12)
	Severe	27	10.0 (5.8, 14.1)	3	7.3 (0.0, 17.3)	44	11.2 (7.5, 15.0)	1.66 (0.81, 3.40)	1.83 (0.54, 6.17)	1.72 (0.85, 3.47)	2.00 (0.63, 6.37)	0.89 (0.52, 1.51)	0.65 (0.16, 2.66)
	Total	85	30.0 (23.8, 36.3)	12	16.7 (10.9, 30.3)	119	28.4 (23.3, 33.6)	0.90 (0.72, 1.14)	0.63 (0.31, 1.29)	1.00 (0.70, 1.42)	0.67 (0.31, 1.43)	1.06 (0.80, 1.39)	0.59 (0.29, 1.18)
Underweight (weight-for-age, WAZ)													
	Moderate	81	29.4 (23.1, 35.8)	20	33.1 (17.8, 48.4)	76	20.6 (15.6, 25.7)	1.77 (1.22, 2.58)	2.12 (1.35, 3.33)	1.73 (1.20, 2.49)	2.21 (1.43, 3.43)	1.43 (1.03, 1.98)	1.60 (0.95, 2.71)
	Severe	65	22.4 (16.9, 28.0)	7	8.0 (2.0, 13.9)	40	10.4 (6.8, 14.0)	2.48 (1.77, 3.49)	1.09 (0.61, 1.97)	2.73 (1.68, 4.43)	1.18 (0.55, 2.54)	2.17 (1.42, 3.32)	0.76 (0.33, 1.75)
	Total	146	51.9 (45.3, 58.4)	27	41.0 (25.6, 56.5)	116	31.0 (25.4, 36.6)	1.48 (1.21, 1.81)	1.53 (1.09, 2.13)	1.76 (1.36, 2.29)	1.89 (1.31, 2.72)	1.67 (1.34, 2.09)	1.32 (0.87, 2.01)

aPR, adjusted prevalence ratio; PR, unadjusted prevalence ratio. Model 1 adjusted by confounders (see the detailed list of confounders by indicator in Table A1). Model 2 adjusted by confounders and population characteristics which differ across flooded and non-flooded communities (Table A1).

**Table 3:** Interaction of the prevalence of total wasting by age groups with number of floods experienced (0,1,2) for adjusted and crude models in 6 to 59 months children in rural Jagatsinghpur district, Odisha, India.

			Repeatedly Flooded (2006, 2008)				Flooded Once (2008)			
		***n***	**aPR (95% CI)**	***P***	**PR (95% CI)**	***P***	**aPR (95% CI)**	***P***	**PR (95% CI)**	***p***
**Flooded *vs.* non-flooded**		764	2.07 (1.44, 2.99)	<0.001	1.79 (1.23, 2.62)	0.002	1.26 (0.69, 2.31)	0.45	1.69 (0.93, 3.07)	0.084
**Child age, months**										
	6–11	85	Reference		Reference		Reference		Reference	
	12–23	185	0.59 (0.38, 0.93)	0.024	0.59 (0.33, 1.04)	0.07	0.59 (0.38, 0.93)	0.024	0.59 (0.33, 1.04)	0.07
	24–35	155	0.28 (0.14, 0.55)	<0.001	0.22 (0.10, 0.48)	<0.001	0.28 (0.14, 0.55)	<0.001	0.22 (0.10, 0.48)	<0.001
	36–47	158	0.16 (0.06, 0.40)	<0.001	0.11 (0.03, 0.39)	<0.001	0.16 (0.06, 0.40)	<0.001	0.11 (0.03, 0.39)	<0.001
	48–59	181	0.66 (0.41, 1.06)	0.09	0.53 (0.29, 0.97)	0.04	0.66 (0.41, 1.06)	0.09	0.53 (0.29, 0.97)	0.04
**Child age x flooding**										
	6–11	85	Reference		Reference		Reference		Reference	
	12–23	185	0.75 (0.42, 1.34)	0.33	0.80 (0.40, 1.61)	0.54	0.54 (0.14, 2.09)	0.37	0.39 (0.10, 1.47)	0.16
	24–35	155	2.00 (0.97, 4.15)	0.06	2.94 (1.26, 6.84)	0.012	2.54 (0.88, 7.32)	0.08	1.94 (0.59, 6.33)	0.27
	36–47	158	4.01 (1.51, 10.63)	0.005	5.62 (1.57, 20.12)	0.008	5.71 (1.78, 18.24)	0.003	5.60 (1.25, 25.15)	0.024
	48–59	181	0.88 (0.52, 1.51)	0.65	1.19 (0.60, 2.37)	0.61	1.57 (0.71, 3.53)	0.27	1.46 (0.60, 3.52)	0.4

aPR, adjusted prevalence ratio; PR, (unadjusted) prevalence ratio. aPR models adjusted by confounders (see in Table A1).

Our findings show that in both exposure groups, communities flooded once or twice, the prevalence of child wasting was substantially increased (relative to those non-flooded). In contrast, being repeatedly exposed to floods proved to have additionally negative effects on these children. This study makes novel contributions to virtually unexplored aspects of the impacts on nutritional health of the children living in flood prone rural areas. First, this evidence improves the understanding of the role of repeated flooding on children’s nutritional status, mainly by showing an increased likelihood of severe wasting in recurrently flooded children. Second, we showed that the youngest children (0–12 months) are particularly vulnerable to the nutritional impacts of floods, which supports previous findings from studies in India, Peru and Ecuador [7,34,35].

Overall, the available literature has pointed to growth failure as the dominant problem in post-flood settings [6,7,34,36]. One study on 180 children after 1998 floods in rural Bangladesh found reductions in the prevalence of wasting from the crisis period (17%) to the onset of the emergency response (12%) [37] but did not investigate the impacts on children’s health after four months. In another study researching the same flood event in Bangladesh, Del Ninno and Lundberg [6] investigated 237 children in three waves up to 15 month post-flood but only found a worsening in height-for-age, where effects were more severe and lasted longer in the youngest cohort (0–12 months), which is consistent with our findings. The same report however proposed that most children have recovered by 15 months. This was not the case in Odisha by far. Whether weight-for-height or height-for-age are more prevalent after flooding depend on the flood experience of the children’s population. Although a higher prevalence of weight-for-height often express a recent process of acute weight loss, it can also reflects chronic unfavorable conditions [33], which was likely the case here. Typically, a rise in the prevalence of wasting might occur if food is suddenly not available. However, our results suggest that situations in which severe crop losses occur in vulnerable populations might set chronic adverse conditions to access food in sufficient quantity. This food deprivation, combined with other factors, may contribute to weight losses among the affected children and eventually trigger a protracted nutritional crisis [33].

In rural India, a previous community-based survey we conducted in the same study area showed that the prevalence of child wasting one month after 2008 floods were 12.2% and 11.9%, respectively in flooded and non-flooded rural communities. This is additional evidence supporting that the observed differences in the prevalence of wasting are due to the effect of flooding and not to baseline differences in child wasting among populations. In a previous study, growth failure proved to be the dominant problem and was attributed to flooding approximately two years earlier, in August 2006 [7]. One plausible explanation for the difference between indicators is the larger magnitude of the 2008 floods compared to those occurring in 2006, which was corroborated with survey household data [7]. Supporting evidence on this aspect is the lower number of villages affected by floods in 2006 compared to 2008 floods. The exceptional severity of the 2008 floods in our study area might have worsened the living conditions of these communities, with more manifest repercussions on the children’ nutritional status. 

In this study, we found the highest prevalence of severe wasting in children exposed to repeated flood, which showcases an important gap in current knowledge. Given that a nearly two-fold difference in the prevalence of severe wasting was observed in those communities repeatedly flooded relative to those flooded in 2008 (aPR: 1.92; 95% CI: 0.95, 3.86), could repeated exposure to floods increase a child’s susceptibility to severe forms of malnutrition? We did not find much evidence on if and how repeated flooding can impact children’s physical health [5]. Some evidence suggest that the likelihood of mental health problems in children affected by disasters (*i.e.*, tsunamis) is also enhanced by previous traumatic experiences, including exposure to war [38]. However, noting that the risk of death is substantially higher in severely wasted children [39], more focused studies addressing this specific question are urgently needed.

This is the first study showing that flood events can produce protracted nutritional crises, even in communities where floods are commonplace. Our study uses a sound epidemiological design, and our findings can be extrapolated to the young children inhabiting 265 rural villages in India. Only one study using primary data has investigated long-term consequences of floods but they found children to recover by 15 months [6]. In addition, our study points to more severe cases of malnutrition in children from communities repeatedly flooded. Finally, our findings suggest infants as the most vulnerable group to the nutritional impacts of flooding, as confirmed by (scant) earlier research [6,7].

Our findings have implications for policy, especially on the protection of those at higher risk and on tailoring more efficient, evidence-based, responses to these crises. Given that the government relief package, including food, was provided for merely 15 days after the 2008 floods, we strongly suggest to expand this period until new crop yields are harvested by the affected populations. Systematic monitoring of the nutritional status of children, mothers and particularly infants is strongly recommended in post-flood settings, especially in vulnerable populations such as low-resource, and rural settings highly relying on subsistence farming, to tailor an adequate response to these crises in the future.

More research is needed and new routes of research need to be explored. Research has focused mainly in Bangladesh, and more recently in India and Peru, but floods occur every day in other vulnerable countries. Global warming is likely to increase the frequency of floods and our results on health impacts of repeated flooding suggest that additional cumulative effects do exist. Thus, if climate change increases the likelihood of a community to be flooded more than once, the chances of having worse nutritional outcomes for children also may increase as demonstrated by our study. If possible, longitudinal studies should be preferable to ascertain causal links between floods and undernutrition in children. Given that infants are more affected than older child cohorts, we think that jointly assessing the nutritional status of mothers and children would help in understanding why. Empirical investigation of the variables mediating the pathways of flood exposure to undernutrition is urgently required as this is key to understanding how floods impact nutritional health in order to design effective interventions and inform preventive policies. Here again, prospective studies would be helpful.

This study has several strengths. First, this is one of the first population-based epidemiological studies exclusively designed to assess the association of flooding and nutritional status in a large population of children aged 6 to 59 months. We did a census targeting children ranging these ages living in each village which allow us to randomly select children (*n* = 3951). Although this is commonly considered best method it is rarely done in practice as it is time consuming. As the sampled units differed between first stage (villages) and second stage (children) of sampling, our design was not self-weighted. Thus, we calculated selection probabilities at each sampling stage to provide correct weights and ensure representativeness of our estimates for a children population estimated to range from 15,979 to 17,602 children. Second, most cross-sectional studies suffer from the inability to ascertain whether exposures occurred before or after the outcomes measured. In this study, the main flood event occurred around one year before our survey and we obtained estimates of the houses flooded within each village by official sources external to the respondents (OSDMA). Third, we took special care to ascertain from OSDMA, corroborated by survey household data, that no other catastrophic event, except the floods in 2006 and 2008, took place since the 1999 Paradip cyclone. Fourth, response rates were very high and consistent across groups, which mostly removed selection bias in our sample. Fifth, we carefully excluded mediating variables, assessed confounders and variables unbalanced between flooded and non-flooded cohorts. Propensity score analyses were carried out to assess the robustness of our findings.

Our study also presents limitations. First, other nutritional deficiencies might be concomitant to those revealed by this study. Future studies should consider assessing bilateral pitting edema as an indicator of Kwashiorkor but also nutritional deficiencies measured by other methods. Second, we did not assess jointly mothers’ and children’ nutritional status. This might have provided additional insights, especially given our results on infants. Third, our study applies to a very particular setting and our results cannot be easily extrapolated to other populations, such as the urban poor. Fourth, no baseline but a comparison group was provided in this study and given its design a causal link is difficult to establish. However, the inclusion of children from nearby non-flooded villages likely contributed to control for unobserved variables (see Appendix Figure A1). Fifth, out-migration could be a potential source of bias if out-migrants are different from those who remain. However, we analyzed unpublished data on a survey conducted two years after the 2008 floods representing similar communities and found that out-migration rates within households was very low in flood-affected areas (2.3%), and also relative to non-flooded areas (1.0%). This is consistent with 15-year record showing modest migration after flooding in Bangladesh [40].

## 5. Conclusions

In these Indian communities, flood-related undernutition persisted well beyond the typical period of emergency relief. In our specific setting, wasting was more prominent than underweight or stunting to indicate the nutritional stresses experienced by the affected child populations. In addition, we found that not only is the burden of undernutrition greater among the flood-affected children and particularly among infants, but the severity of this condition is significantly higher in repeatedly flooded children. Global climate changes are set to increase flooding both in frequency and severity which will further aggravate this situation, particularly in low-resource settings or rural populations where susbsistence farming is very common [41]. In these contexts, we recommend the following actions. Protracted nutritional response after floods should be seriously considered to counteract the long-term nutritional effects on children. In areas where flooding is a recurrent occurrence, policies for relief should be reviewed to ensure a longer period of support and proper take up by development programs. Particularly attention should be paid to the nutritional health of mothers as the effects are worst amongst infants. Most importantly, preventive action should be put in place to protect mothers and children in flood prone areas. Systematic and long-lasting monitoring of the nutritional status of vulnerable groups in the aftermath of floods (but also pre-flood if possible) might be helpful to tailor the response and food supply in each particular context. Further stringent epidemiological studies that bring evidence on floods and nutrition to inform public health policies within the climate change framework are urgently required.

## Abbreviations

The following abbreviations are used in this manuscript:

aPR: adjusted prevalence ratio
ENA: emergency nutrition assessment
ICDS: Integrated Child Development Scheme
OSDMA: Odisha state disaster mitigation authority
PR: prevalence ratio
PSU: primary sampling unit
SSU: secondary sampling unit
SMART: standardized monitoring and assessment of relief and transitions
UNICEF: United Nations children’s fund

## Appendix A

**Table A1:** Overview of variables with frequencies differing across exposure and plausible confounders of undernutrition indicators in children of rural Jagatsinghpur district, Odisha, India.

	Flooded *vs.* Non-Flooded	Confounders (*p* ≤ 0.2)								
**Variables**		**Wasting**			**Stunting**			**Underweight**		
**Child characteristics**		Moderate	Severe	Total	Moderate	Severe	Total	Moderate	Severe	Total
Child female	NS	No	Yes	Yes	Yes	No	Yes	No	No	No
Mean birthweight, g	NS	Yes	Yes	Yes	No	Yes	Yes	No	Yes	Yes
Child age, months	*p* ≤ 0.2	Yes	Yes	Yes	Yes	Yes	Yes	Yes	Yes	**No**
**Underlying Causes**										
Care of mother and children										
Mean age at marriage of the mother, y	*p* ≤ 0.2	**No**	**No**	**No**	**No**	**No**	**No**	Yes	Yes	Yes
Mean age of the mother at first delivery, y	*p* ≤ 0.2	Yes	Yes	Yes	Yes	**No**	Yes	**No**	Yes	**No**
Mean age of the mother at birth of child, y	NS	Yes	Yes	No	No	No	Yes	Yes	No	Yes
Mean age of the father at birth of child, y	NS	No	Yes	Yes	Yes	No	No	No	Yes	No
Two or more children underfive eating from same kitchen	NS	No	No	No	No	No	No	No	No	No
Mother was a member of self-help group/Mahila Mandal	NS	Yes	Yes	No	No	No	No	No	No	No
Mean number of mother‘s conceptions	NS	Yes	Yes	No	Yes	No	Yes	Yes	No	Yes
Mother had one or more miscarriages, stillbirths	NS	No	No	Yes	Yes	Yes	Yes	No	No	No
Child breastfed at least 2 years	NS	No	No	No	No	No	No	No	Yes	No
Supplementary feeding started after 6 months of child‘s age	NS	Yes	Yes	No	No	Yes	Yes	No	Yes	No
Child breastfed at least 2 years and supplemented after 6 months of age	*p* ≤ 0.2	Yes	**No**	Yes	No	**No**	**No**	**No**	**No**	**No**
Mother washes hands with soap before feeding child	*p* ≤ 0.2	**No**	**No**	**No**	**No**	**No**	**No**	**No**	**No**	**No**
Mother washes hands with soap before preparing food for the family	*p* ≤ 0.2	Yes	**No**	Yes	**No**	**No**	**No**	**No**	**No**	**No**
Mother washes hands with soap before lunch	NS	No	Yes	Yes	Yes	Yes	No	No	No	No
Mother washes hands with soap after attending the child who defecated	NS	Yes	No	Yes	Yes	Yes	Yes	Yes	No	Yes
Child received BCG vaccine	NS	No	Yes	Yes	No	Yes	No	Yes	Yes	Yes
Child received measles vaccine	NS	No	Yes	No	No	Yes	Yes	No	Yes	Yes
Child received all three doses of polio vaccine	NS	Yes	Yes	Yes	Yes	No	Yes	No	No	No
Child received all three doses of B-hepatitis vaccine	NS	No	Yes	No	No	Yes	No	Yes	No	Yes
Child received all three doses of DPT vaccine	NS	Yes	Yes	No	Yes	Yes	Yes	Yes	Yes	No
Public health										
Improved latrine (before floods)	*p* ≤ 0.2	**No**	**No**	**No**	**No**	Yes	**No**	Yes	**No**	**No**
Improved drinking water (before floods)	*p* ≤ 0.2	**No**	**No**	**No**	**No**	**No**	**No**	**No**	Yes	**No**
Mother gave birth at hospital	NS	No	No	No	No	No	No	Yes	No	Yes
Number of individuals eating from same kitchen	*p* ≤ 0.2	Yes	Yes	Yes	Yes	**No**	Yes	**No**	Yes	Yes
Capital										
Household did borrow money (loan, credit, micro-credit)	NS	No	No	No	Yes	No	Yes	Yes	Yes	No
Maternal education	*p* ≤ 0.2	Yes	Yes	Yes	Yes	Yes	Yes	**No ^1^**	Yes	Yes
Paternal education	*p* ≤ 0.2	Yes	Yes	Yes	**No**	Yes	Yes	Yes	Yes	Yes
Land ownership, hectare	*p* ≤ 0.2	**No**	Yes	**No**	Yes	Yes	Yes	**No**	Yes	Yes
**Basic causes**										
Religion	NS	Yes	Yes	Yes	Yes	Yes	Yes	Yes	Yes	Yes
Caste	*p* ≤ 0.2	Yes	Yes	Yes	Yes	Yes	Yes	Yes	Yes	Yes

^1^ Model did not converge using mother education and this variable was excluded. NS, non-significant (*i.e.*, *p* > 0.2).

**Table A2:** Final model results after implementation of propensity score matching to the sample of children inhabiting repeatedly flooded and non-flooded villages.

		Model 1		Model 2	
		**aPR (95% CI)**	***p***	**aPR (95% CI)**	***p***
Flooded *vs.* non-flooded		1.93 (1.34, 2.79)	<0.001	2.22 (1.45, 3.40)	<0.001
Child age, months					
	6–11	Reference		Reference	
	12–23	0.46 (0.25, 0.87)	0.02	0.60 (0.32, 1.14)	0.12
	24–35	0.25 (0.11, 0.57)	0.009	0.31 (0.14, 0.71)	0.006
	36–47	0.13 (0.04, 0.51)	0.003	0.15 (0.04, 0.54)	0.004
	48–59	0.65 (0.39, 1.07)	0.095	0.70 (0.36, 1.35)	0.29
Child age x flooding					
	6–11	Reference		Reference	
	12–23	0.92 (0.44, 1.92)	0.83	0.73 (0.36, 1.50)	0.40
	24–35	2.31 (0.94, 5.65)	0.07	1.99 (0.82, 4.83)	0.13
	36–47	5.16 (1.31, 20.35)	0.02	4.29 (1.19, 15.48)	0.03
	48–59	0.95 (0.55, 1.64)	0.84	0.88 (0.45, 1.71)	0.70

Model 1 shows the results of the final model after conducting propensity scores matching (with nearest neighbor method) on 33 variables described in Table 1. Model 2 shows the results of the final model after conducting propensity scores matching (with nearest neighbor method) on 22 variables which were either plausibly confounders or were unbalanced between flooded and non-flooded communities (Table A1).

## Appendix B

**Table B1:** STROBE Statement—Checklist of items that should be included in reports of *cross-sectional studies*.

		Item No.	Recommendation
Title and abstract		1✓ p1–2	(*a*) Indicate the study’s design with a commonly used term in the title or the abstract
			(*b*) Provide in the abstract an informative and balanced summary of what was done and what was found
Introduction			
Background/rationale		2✓ p3–4	Explain the scientific background and rationale for the investigation being reported
Objectives		3✓ p4	State specific objectives, including any prespecified hypotheses
Methods			
Study design		4✓ p4–5	Present key elements of study design early in the paper
Setting		5✓ p6	Describe the setting, locations, and relevant dates, including periods of recruitment, exposure, follow-up, and data collection
Participants		6✓ p5–8	(*a*) Give the eligibility criteria, and the sources and methods of selection of participants
Variables		7✓ p5, 8–9	Clearly define all outcomes, exposures, predictors, potential confounders, and effect modifiers. Give diagnostic criteria, if applicable
Data sources/measurement		8✓ p8–9	For each variable of interest, give sources of data and details of methods of assessment (measurement). Describe comparability of assessment methods if there is more than one group
Bias		9✓ p5, 9, 10 and SOM	Describe any efforts to address potential sources of bias
Study size		10✓ p7	Explain how the study size was arrived at
Quantitative variables		11✓ p5, 8 and tables	Explain how quantitative variables were handled in the analyses. If applicable, describe which groupings were chosen and why
Statistical methods		12✓ p9–10	(*a*) Describe all statistical methods, including those used to control for confounding
			(*b*) Describe any methods used to examine subgroups and interactions
			(*c*) Explain how missing data were addressed
			(*d*) If applicable, describe analytical methods taking account of sampling strategy
			(*e*) Describe any sensitivity analyses
Results			
Participants		13✓ p10	(a) Report numbers of individuals at each stage of study—e.g., numbers potentially eligible, examined for eligibility, confirmed eligible, included in the study, completing follow-up, and analysed
			(b) Give reasons for non-participation at each stage ✓ Figure 1
			(c) Consider use of a flow diagram ✓ Figure 1
Descriptive data		14✓ p10, table 1	(a) Give characteristics of study participants (e.g., demographic, clinical, social) and information on exposures and potential confounders
			(b) Indicate number of participants with missing data for each variable of interest
Outcome data		15✓ p10, table 2	Report numbers of outcome events or summary measures
Main results		16✓ p10–11, tables 2–3 tables 1–3 p5 n/a	(*a*) Give unadjusted estimates and, if applicable, confounder-adjusted estimates and their precision (eg, 95% confidence interval). Make clear which confounders were adjusted for and why they were included ✓ (p10)
			(*b*) Report category boundaries when continuous variables were categorized
			(*c*) If relevant, consider translating estimates of relative risk into absolute risk for a meaningful time period
Other analyses	11✓ p7		Report other analyses done—e.g., analyses of subgroups and interactions, and sensitivity analyses
Discussion			
Key results	18✓ p12		Summarise key results with reference to study objectives
Limitations	19✓ p14–15		Discuss limitations of the study, taking into account sources of potential bias or imprecision. Discuss both direction and magnitude of any potential bias
Interpretation	20✓ p14–16		Give a cautious overall interpretation of results considering objectives, limitations, multiplicity of analyses, results from similar studies, and other relevant evidence
Generalisability	21✓ p15–16		Discuss the generalisability (external validity) of the study results
Other information			
Funding	22✓ p1		Give the source of funding and the role of the funders for the present study and, if applicable, for the original study on which the present article is based
